# Valproic acid inhibits cell growth in both MCF-7 and MDA-MB231 cells by triggering different responses in a cell type-specific manner

**DOI:** 10.1186/s12967-023-04015-8

**Published:** 2023-03-02

**Authors:** Francesca Giordano, Alessandro Paolì, Martina Forastiero, Stefania Marsico, Francesca De Amicis, Mariangela Marrelli, Giuseppina Daniela Naimo, Loredana Mauro, Maria Luisa Panno

**Affiliations:** Department of Pharmacy, Health and Nutritional Sciences, University of Calabria, 87036 Rende, Italy

**Keywords:** Breast cancer, Valproic acid, ROS, Apoptosis, Inflammation

## Abstract

**Background:**

Breast cancer is the second leading cause of death among women after lung cancer. Despite the improvement in prevention and in therapy, breast cancer still remains a threat, both for pre- and postmenopausal women, due to the development of drug resistance. To counteract that, novel agents regulating gene expression have been studied in both hematologic and solid tumors. The Histone Deacetylase (HDAC) inhibitor Valproic Acid (VA), used for epilepsy and other neuropsychiatric diseases, has been demonstrated a strong antitumoral and cytostatic activity. In this study, we tested the effects of Valproic Acid on the signaling pathways involved in breast cancer cells viability, apoptosis and in Reactive Oxygen Species (ROS) production using ER-α positive MCF-7 and triple negative MDA-MB-231 cells.

**Methods:**

Cell proliferation assay was performed by MTT Cell cycle, ROS levels and apoptosis were analyzed by flow cytometry, protein levels were detected by Western Blotting.

**Results:**

Cell treatment with Valproic Acid reduced cell proliferation and induced G0/G1 cell cycle arrest in MCF-7 and G2/M block in MDA-MB-231 cells. In addition, in both cells the drug enhanced the generation of ROS by the mitochondria. In MCF-7 treated cells, it has been observed a reduction in mitochondrial membrane potential, a down regulation of the anti-apoptotic marker Bcl-2 and an increase of Bax and Bad, leading to release of cytochrome C and PARP cleavage. Less consistent effects are recorded in MDA-MB-231 cells, in which the greater production of ROS, compared to MCF-7cells, involves an inflammatory response (activation of p-STAT3, increased levels of COX2).

**Conclusions:**

Our results have demonstrated that in MCF-7 cells the Valproic Acid is a suitable drug to arrest cell growth, to address apoptosis and mitochondrial perturbations, all factors that are important in determining cell fate and health. In a triple negative MDA-MB 231 cells, valproate directs the cells towards the inflammatory response with a sustained expression of antioxidant enzymes. Overall, the not always unequivocal data between the two cellular phenotypes indicate that further studies are needed to better define the use of the drug, also in combination with other chemotherapy, in the treatment of breast tumors.

**Supplementary Information:**

The online version contains supplementary material available at 10.1186/s12967-023-04015-8.

## Background

Epigenetic processes, which affect DNA accessibility, impact on multifactorial function in normal and transformed cells. [1, 2]. In particular, DNA methylation and histone modifications, through the enzymatic action of histone acetyltransferase (HAT) and histone deacetylase (HDAC) modulate a large number of cellular responses and play a relevant role in tumorigenicity [3].

Several studies have shown that in many tumor types, high HDAC enzyme expressions or complete loss of histone acetylation well correlate with a poorer prognosis [4, 5]. Based on structural homology, HDACs have been grouped into 4 major classes. Class I and II, which include HDACs 1, 2, 3 and 8, are recognized as critical for tumorigenesis. Numerous studies have suggested a correlation between the expression levels of class I HDACs and breast cancer subtypes, aggressiveness and the presence of ER, PR and HER-2. HDACs class II enzymes are involved in breast cancer progression and response to therapeutic treatments [6–9].

Numerous HDAC inhibitors have been studied and several of them have shown to influence growth, apoptosis and invasiveness in cancer cells both *in vitro* and *in vivo*. In breast cancer, the genes that regulate these events are often hypermethylated, such as Cyclin D2, thus causing a perturbation of the cell cycle and tumor progression [10–16].

Indeed, some HDAC inhibitors, alone or in combination with other chemotherapeutic agents, are used in many clinical trials for both hematologic and solid tumors [17]. Valproic acid (VA), a short-chain fatty acid, which has been used for the past two decades in the treatment of epilepsy and other neuropsychiatric diseases, is considered a class I HDAC inhibitor with strong antitumoral activity [18].

Other studies have shown that VA impairs tumor progression through inhibition of cell proliferation, cell cycle regulation, DNA repair, and apoptosis [19, 20], as well as it can alter differentiation process and angiogenesis in prostatic tumoral cells [21, 22].

VA has also been studied in breast cancer, which represents the second leading cause of death among women after lung cancer [23]. Despite the increase and improvement of preventive screening, breast cancer still remains a threat to both pre- and postmenopausal women, due to the varied histopathological features and hormonal status of tumor that determine a different effectiveness of response to treatments. The HDAC inhibitor VA showed anti tumoral effects according to the dose and cell type used. Regarding breast cancer, VA has been reported to affect hormone receptor and to up-regulate the expression of cyclin dependent kinase inhibitors, such as p21 and p57, with a consequent arrest of cell progression [24].

In accordance with the above findings, depending on the receptor status, breast cancers are treated with surgery and adjuvant chemotherapy, which includes endocrine-based agents, such as SERMS (tamoxifen), SERDs (fulvestran) and aromatase inhibitors (letrazole and anastrazole). However, it is known that over time, breast cancer often develops resistance to drug therapy, that can be traced to several mechanisms, such as mutations in the estrogen receptor, altered expression of the same receptor (epigenetic modifications), changes in the levels of metabolic enzymes involved in hormone synthesis and aberrant activation of signal transduction pathways (up- regulation of PI3K, MAPK, CDKs) [25, 26].

To overcome the mechanisms of resistance, novel agents, that negatively act on target molecules important in cell growth, such as EGF receptor (lapatinib) and CDK4/6 receptor (ribociclib and palbociclib) inhibitors, have been developed and used in the clinic in conjunction with endocrine therapy and in particular, with the aromatase inhibitor letrazol.

VA has emerged as an active compound in the treatment of resistant or metastatic breast cancer associated with chemotherapy or endocrine therapy [27]. Indeed, the association of VA and cisplatin induces apoptosis in breast cancer cells and this improves the efficacy of the response compared to monotherapy alone [28]. Interestingly, synergy between VA and SERMs leads to a better breast cancer prognosis. However, the anticancer activity and the specificity of VA need to be further investigated, given the results not always unique in this context.

Regarding breast cancer cells, it has been reported that VA shows more satisfactory responses in hormonal receptor positive cells than those receptor-negative. This highlights that the action of the molecule is aimed at different target signaling pathways that account for the cell-type specific effect. Considering the use of VA, as promising anticancer agent, we further focused our attention on signaling pathways mainly involved in breast cancer cells viability, using ER-α positive MCF-7 and triple negative (ER-α, PR, HER2) MDA-MB-231 cells.

### Methods reagents

Valproic Acid sodium salt was purchased from Sigma-Aldrich (Merck)(Cod. p4543-10G).

### Cell culture

Human breast cancer epithelial cell line MCF-7 (estrogen receptor (ERα-positive) and triple-negative human breast cancer cell line MDA-MB-231 (ER-, PR-, HER-2-negative) were cultured in DMEM/F12 containing 10% fetal bovine serum (FBS) (Life Technologies) at 37 °C with 5% CO2 air. Human normal breast epithelial cell line MCF-10A was grown in DMEM-F12 medium containing 5% horse serum (Life Technologies).

### Cell viability assay

MCF-7, MDA-MB-231 and MCF-10A cells (5 × 10^3^ cells/mL) were grown in 96well plates and incubated to allow attachment. Then cells were incubated in phenol red free medium (PRF-SFM DMEM/F12) for 24 h and treated with VA at different concentrations (0.5, 1, 1.5, 2, 2.5, 3, 3.5 mM) for 48 and 72 h. At the end of incubation 100 µl of 2 mg/ml MTT (3-[4,5-dimethylthiazol-2-yl]- 2,5-diphenyl tetrazolium) (Sigma-Aldrich, Merck) was added to each well and incubated at 37 °C for 4 h. Subsequently, the medium was removed and 100μl/well DMSO was added to solubilize the formazan. Finally, the optical density of the soluble formazan was read at 570 nm with a plate reader (Multiskan EX, Thermofisher System).

### Cell cycle analysis

To determine cell cycle distribution analysis, MCF-7 and MDA-MB-231 cells were cultured in regular medium in 6 well plates and shifted in medium without serum for 24 h. Next, both cells were exposed to treatments with 2 mM of VA for 24 and 48 h. At the end of incubation, the cells were pelleted, once washed with PBS and fixed in 50% methanol overnight at  − 20 °C and stained with a solution containing 50 μg/ml propidium iodide (PI), 20 U/ml RNAse-A and 0.1% Triton (Merck Life Science, Milan, Italy). Cell phases were estimated as a percentage of a total of 10 000 events. The DNA content was measured using a FACScan flow cytometer (Becton Dickinson, Mountain View, CA, USA) and the data acquired using CellQuest software. Cell cycle profiles were determined using ModFit LT [29].

### Annexin V/PI assay

The Annexin V-FITC Kit-Apotosis Detection (Beckman Coulter) was used to perform the annexin V/PI assay. The breast cancer cells (2 × 10^5^/well in 2 ml of medium) were seeded in 6-well plates. Next, the cells were treated with VA for 12 and 24 h. At the end of the treatment the cells were collected with trypsin, centrifuged at 1000/1200 rpm for 5 min, resuspended in PBS and counted. Cells (1 × 10^6^ cells/ml of buffer) were resuspended in 1 × binding buffer provided by the kit. 100 µl (containing 10^5^ cells) were transferred into a tube and incubated with 2 µl of Annexin V and 5 µl of PI for 15 min in the dark at room temperature. At the end of the incubation, 400 µl of Binding Buffer 1 × were added to each tube and the samples were analyzed by FACScan flow cytometer (Becton Dickinson, Mountain View, CA, USA) and the data were acquired using CellQuest software.

### Immunoblotting analysis

MCF-7 and MDA-MB-231 cells were grown to 70–80% confluence and treated in PRF-SFM DMEM/F12, with VA for 24 and 48 h. At the end of each treatment, cells were lysed with 500 μl of RIPA buffer (50 mM Tris–HCl, 150 mM NaCl, 1% NP-40, 0.5% sodium deoxycholate, 2 mM sodium fluoride, 2 mM EDTA, 0.1% SDS) with protease inhibitors (1.7 mg/ml aprotinin, 1 mg/ml leupeptin, 200 mmol/l phenylmethylsulfonyl fluoride, 200 mmol/l sodium orthovanadate and 100 mmol/lsodium fluoride; Sigma-Aldrich, Merck).

Equal amounts of proteins were resolved on 8% and 12% SDS/polyacrylamide gel, transferred to a nitrocellulose membrane and probed with primary antibodies against: Cyclin D1, Cyclin B1, p21, p-38, p-ERK (Invitrogen, Thermo Fisher Scientific); Bcl2, Bad, p-Bad, cythocrome C, Survivin, COX2, Catalase, SOD1, ERK2, Actin (Santa Cruz Biotechnology, DBA, Milan, Italy); p-STAT3, STAT3, JNK and p-p38 (Cell Signaling Technology, Euroclone, Milan, Italy); p-JNK, Bax (Bios, Massachusetts, USA); GAPDH (ProteinTech).

The antigen-antibody complex was detected by incubation of the membranes with peroxidase- coupled goat anti-mouse or goat anti-rabbit antibodies and then revealed using the chemiluminescent substrate for Western Blotting, ECL System (Amersham Pharmacia, Buckinghamshire UK) [29].

### Evaluation of mitochondrial mass and mitochondrial membrane potential

Mitochondrial mass and membrane potential were measured by FACS analysis of cells stained with MitoTracker^®^ Deep Red (mitochondrial mass evaluation) or MitoTracker^®^ Orange CM-H2TMRos (mitochondrial membrane potential evaluation) (Life Technologies), as previously reported [30].

Briefly, MCF-7 and MDA-MB-231 cells were plated and treated or untreated in PRF-SFM-DMEM/F12 for 48 h with VA. The cells were collected and incubated with MitoTracker staining solution (10 nM final concentration in PBS) for 30–60 min at 37 °C. Cells were then harvested, re-suspended in PBS and analyzed by flow cytometry (CytoFLEX Beckman, Beckman Coulter, Milan, Italy). Data analysis was performed using CytExpert Beckman Coulter software (Beckman Coulter, Milan, Italy).

### Reactive oxygen species (ROS) assessment

ROS were quantified using the chloromethyl derivative of 2′,7′-dichlorodihydrofluorescein diacetate (CM-H2DCFDA, Thermo Fisher Scientific, Waltham, MA, USA), as the manifacturer’s recommendations. Briefly, MCF-7 and MDA-MB-231 cells were plated and treated or untreated in PRF- SFM-DMEM/F12 for 24 and 48 h with 2 mM of VA. Then, the treated cells were rinsed with PBS, harvested, resuspended in 5 μM CM-H2DCFDA, a fluorescent probe used as an indicator for ROS, in PBS and incubated at 37 °C, for 30–40 min. Subsequently, the stained cells were harvested by centrifugation and maintained in a fresh medium and incubated at 37 °C for 20 min. The cells were analyzed by flow cytometry (CytoFLEX Beckman, Beckman Coulter, Milan, Italy). Data analysis was performed using CytExpert Beckman Coulter software (Beckman Coulter, Milan, Italy).

### Statistical analysis

Data obtained from multiple independent experiments are expressed as the mean ± standard deviation (SD). Data were analyzed for statistical significance using the Bonferroni post-test. Student’s t test for unpaired data (2-tailed) was used to test the probability of significant differences between two groups of samples. Differences were considered significant when p ≤ 0.05 and p ≤ 0.005. Statistical tests were performed using GraphPad Prism software (GraphPad Software, La Jolla, CA, USA).

## Results

### Valproic acid inhibits breast cancer cell proliferation

**Fig. 1:**
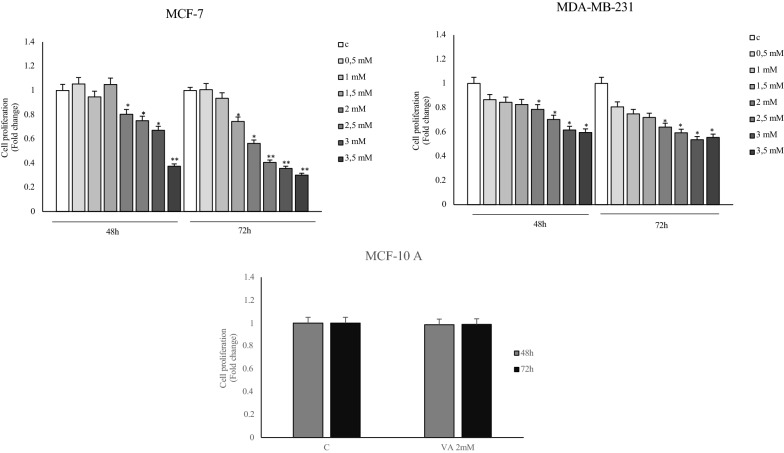
Effects of VA on breast cancer cell growth. MTT growth assays, in MCF-7 and MDA-MB-231 cells treated with increasing doses (0.5, 1, 1.5, 2, 2.5, 3 and 3.5 mM) and in MCF-10 A normal cells treated with 2 mM of VA for 48 and 72 h. Cell proliferation is expressed as fold change ± S.D. relative to control (**C**) cells and is representative of three different experiments each performed in triplicate. * p < 0.05 and **p < 0.005

We evaluated the impact of the Valproic Acid (VA) on breast cancer cell viability using both ER-α positive MCF-7 and triple negative (ER-α, PR, HER2) MDA-MB-231 breast cancer cell lines. For this purpose, the effects of increasing doses (0.5, 1, 1.5, 2, 2.5, 3 and 3.5 mM) for 48 and 72 h of the Valproic Acid were tested on cell viability by using MTT assays. The results obtained showed a reduction in cell survival, in a dose and time dependent manner, in MCF-7 and MDA-MB231 cells, compared to control (Fig. 1). Considering that the 2 mM dose of VA gave the first significant result on cell proliferation, this concentration was tested in normal breast MCF-10A cell line. The same dose was chosen for subsequent experiments. The data reported on Fig. 1, shown no effect in MCF-10 cells, suggesting a specific action of VA only in transformed cells.

**Fig. 2:**
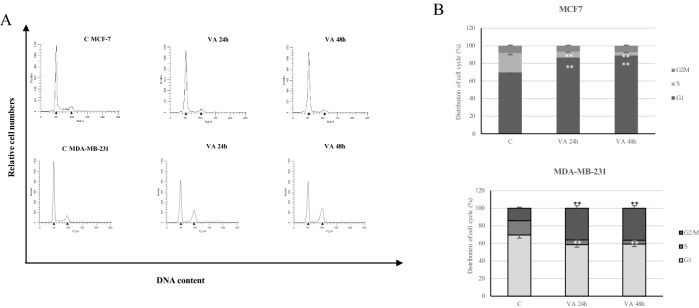
Effects of VA on cell cycle distribution in breast cancer cells. **A** MCF-7 and MDA-MB-231 cells were untreated C or treated with VA as indicated for 24 and 48 h, stained with propidium iodide (PI) and analyzed on a FACScan flow cytometer. **B** Quantitative analysis of percentage gated cells at G0/G1, S and G2/M phases were shown. **p < 0.005 vs Control (C)

Subsequently, we performed flow cytometric cell cycle analysis. As shown in Fig. 2, in MCF-7 cells treated with VA for 24 and 48 h we observed an increase in G1 phase of 86.56% and 89.2%, respectively, compared to control (69.93%). Concomitantly, the S phase is reduced passing from 22.01 % of control, to 6.8% and 3.43% after 2 and 48 h of treatment. Differently, in MDA-MB-231 cells, the VA induces G2/M phase arrest, causing an increase in the percentage of cells in this phase of 36.7% and 36.26% (24 and 48 h) compared to control (14.12%). As in MCF-7, the VA treatment in MDA-MB-231 cells confirms a consistent reduction of the S phase.

**Fig. 3:**
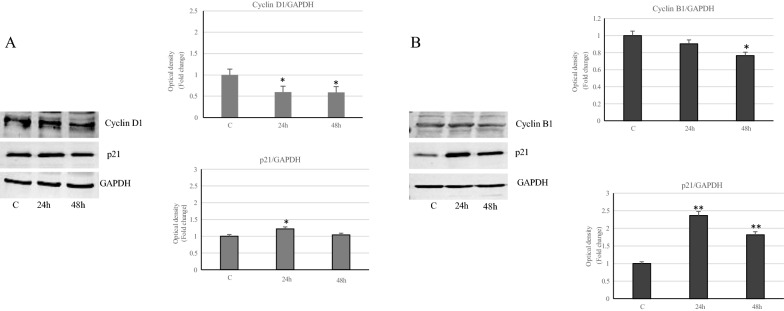
VA effects on cell cycle regulatory proteins. Immunoblot of Cyclin D1 and p21 expression levels in MCF-7 cells (**A**) and Cyclin B1 and p21 in MDA-MB-231 cells (**B**) treated with VA for 24 and 48 h. GAPDH was used as a protein loading control. Histograms represent the mean ± SD of three experiments in which band intensities were evaluated in terms of arbitrary units of optical density (OD) and expressed as fold change relative to control *p<0.05 and **p<0.005 vs Control (C).

In order to elucidate the mechanisms by which the VA induced cell cycle arrest, we evaluated the change in expression levels of specific cell cycle regulatory proteins. ìCyclin D1, which allows cells to progress from G1 phase to S phase, showed a significant reduction in MCF-7 treated cells, suggesting in this latter condition a block in G0/G1, while in MDA-MB-231 cells VA-treatment induced a decreased expression of cyclin B1 (Fig. 3). Analogously, Western blotting analysis showed increased p21 expression levels in both cell lines compared to control, particularly in MDA-MB-231 cells (Fig. 3).

### VA treatment induces ROS production and impairs the mitochondrial function in breast cancer cells

**Fig. 4:**
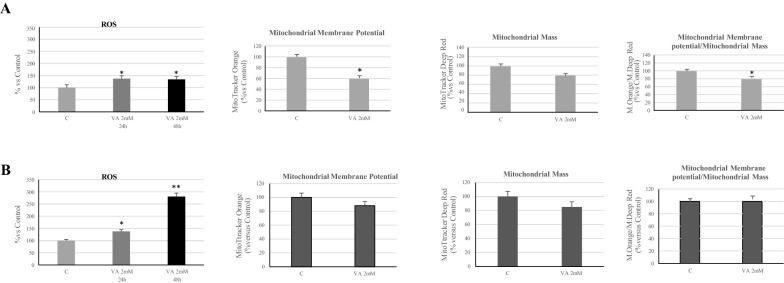
Action of VA on ROS production and mitochondrial function. Intracellular ROS levels, Mitochondrial Membrane Potential and Mitochondrial Mass were analyzed in MCF-7 and MDA-MB-231 cells. The ratio between mitochondrial membrane potential and mitochondrial mass is also displayed. Untreated cells (C) were used as negative control. Values represent the mean of three triplicate independent experiments *p < 0.05 vs C. **p < 0.005 vs C

To asses ROS production, MCF-7 and MDA-MB-231 cells were incubated with 5μM of the chloromethyl-20,70-dichlorofluorescein diacetate probe, conjugated to FITC (CM-H2DCFDA, Molecular Probe, Invitrogen) and analyzed by flow cytometry. Our results showed a marked induction of ROS species in both breast cancer cells undergoing treatment (Fig. 4). In particular, in MDA-MB-231 cells, the prolonged incubation with VA until 48 h, induces a more consistent production of ROS compared to MCF-7 cells.

Next, we evaluated whether Valproic Acid could affect the mitochondrial number and function. To determine the electrochemical potential across the mitochondrial membrane and the change in the mitochondrial mass we used the MitoTracker Orange and the MitoTracker Deep Red probes, respectively. In MCF-7 cells, treated with Valproic Acid, a reduction in mitochondrial membrane potential and in mitochondrial mass has been observed. The ratio of mitochondrial membrane potential/mass indicated, consequently, an alteration of mitochondrial function (Fig. 4A). Under the same experimental conditions, less consistent effects were observed in MDA-MB-231 cells on mitochondrial function (Fig. 4B).

### VA induces the intrinsic apoptosis pathway

**Fig. 5:**
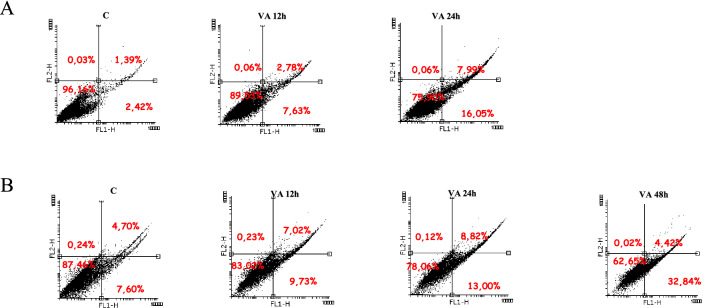
VA in MCF-7 cells induces apoptosis. MCF-7 (**A**) and MDA-MB-231 (**B**) cells after treatment with valproic acid at the indicated times were stained with Annexin V-FITC and PI. Apoptosis was assessed by flow cytometry. The four quadrants represent living cells (lower left, AnnexinV-/PI-), early apoptotic (lower right, Annexin V+/PI-), late apoptosis (upper right, Annexin V+/PI+) or necrotic (upper left, Annexin V-/PI+) stages. Values represent the percentages of each quadrant.

The main morphological changes of the apoptotic process are chromatin condensation and abnormalities in the structure and stability of cell membrane components, such as translocation of phosphatidylserine to the cell surface [31, 32]. Morphological changes in the cell membranes of VA-treated MCF-7 cells were analyzed at 12 and 24 h by staining the cells with a combination of annexin V, FITC-conjugated and propidium iodide (PI), which discriminates viable cells from those in early or in late apoptosis phase and in necrosis. As shown in Fig. 5A, we observed a shift in the percentage of apoptotic cells in early phase (lower right quadrant) of 7.63% and 16.05%, respectively after 12 and 24 h of VA treatment, compared to untreated cells (2.42%). The percentage of apoptotic cells in the late stage increased from 1.39% in untreated cells to 2.78% at 12 h and 7.99% after 24 h of VA treatment. In MDA-MB-231 cells, it was not observed a substantial increase of apoptotic cells in early phase, while this was reveled only when the treatment was prolonged up to 48 h (Fig. 5 B).

**Fig. 6:**
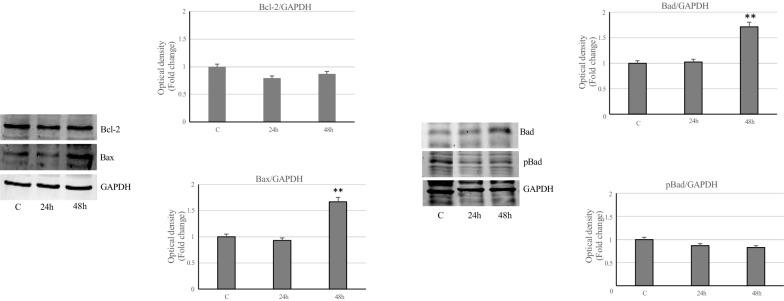
Effects of VA on the key proteins of apoptosis in MCF-7 cells. Immunoblots of Bcl-2, Bax, Bad and pBad protein levels in MCF-7 cells untreated or treated with valproic acid for 24 and 48 h. GAPDH was used as a protein loading control. Histograms represent the mean ± SD of three separate experiments in which band intensities were evaluated as optical density (OD) and expressed as fold change relative to control. **p < 0.005 vs Control (C)

**Fig. 7:**
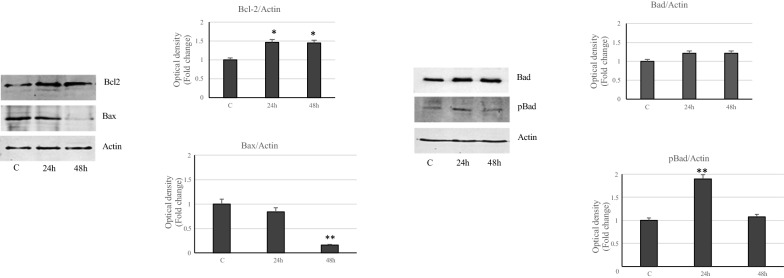
Effects of VA on the key proteins of apoptosis in MDA-MB-231 cells. Immunoblots of Bcl-2, Bax, Bad and pBad protein levels in MDA-MB-231 cells untreated or treated with VA for 24 and 48 h. Actin was used as a protein loading control. Histograms represent the mean ± SD of three separate experiments in which band intensities were evaluated as optical density (OD) and expressed as fold change relative to control. *p < 0.05 and **p < 0.005 vs. Control (C)

Then, we have focused on the Bcl2 family of proteins involved in the regulation of apoptotic process. Immunoblotting analysis showed reduced levels of antiapoptotic Bcl-2 protein in MCF-7 breast cancer cells treated with VA compared to control, while increased expression of pro-apoptotic Bax and Bad, was evidenced with the prolongation of incubation (Fig. 6). The phosphorylation of Bad protein on ser 112, which inhibits the pro-apoptotic response is reduced in MCF-7 treated-cell, compared to control cells (Fig. 6). Conversely, MDA-MB-231 cells treated with VA react to apoptosis, showing an up-regulation of survival protein Bcl-2, decreased Bax levels with an increase ofBad, even if not significant, compared to control (Fig. 7). Furthermore, Bad phosphorylation, increased transiently at 24 h of treatment in MDA-MB-231 cells, confirming in these oncogenic phenotypes a different response to the drug than in MCF-7 cells (Fig. 7).

**Fig. 8:**
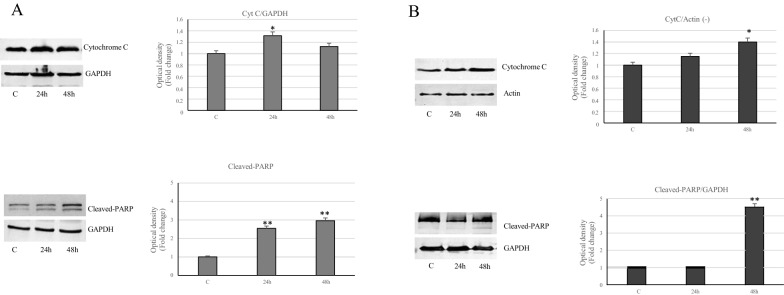
Expression of cytochrome C and PARP in breast cancer treated-cells. Immunoblots of cytochrome C and cleaved-PARP protein levels in MCF-7 (**A**) and MDA-MB-231 (**B**) cells untreated or treated with VA for 24 and 48 h. GAPDH and actin were used as a protein loading control. Histograms represent the mean ± SD of three separate experiments in which bandi ntensities were evaluated as optical density (OD) and expressed as fold change relative to control *p<0.05 and **p<0.005 vs Control (C)

**Fig. 9:**
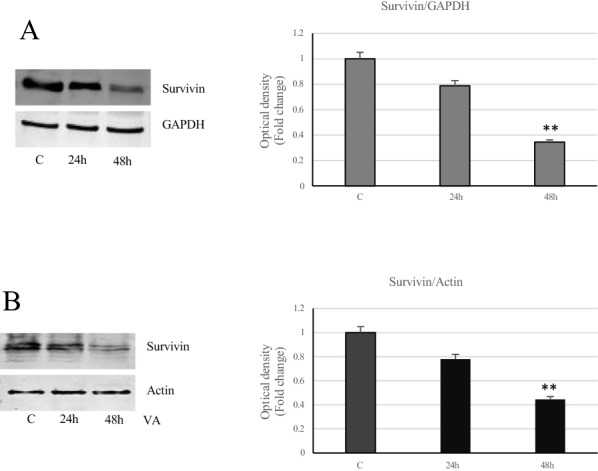
: Role of VA on survivin in breast cancer cells. Immunoblots of survivin levels in MCF-7 (**A**) and MDA-MB-231 (**B**) cells untreated or treated with VA for 24 and 48 h. GAPDH and Actin were used as a protein loading control. Histograms represent the mean ± SD of three separate experiments in which bandi ntensities were evaluated as optical density (OD) and expressed as fold change relative to control. **p < 0.005 vs Control (C)

Since Bcl-2 family proteins control apoptosis, by regulating outer mitochondrial membrane permeabilization, we focused our to cytochrome C and poly (ADP-ribose) polymerase (PARP). Immunoblotting analysis showed increased levels of cytochrome C and of the proteolytic form of PARP in MCF-7 and MDA-MB231 cancer cells, treated with VA, especially at 48 h (Fig. 8). Furthermore, under the same experimental conditions, a reduction of survivin protein expression has been revealed in both cell types (Fig. 9).

### Effects of VA on MAPK pathways

**Fig. 10:**
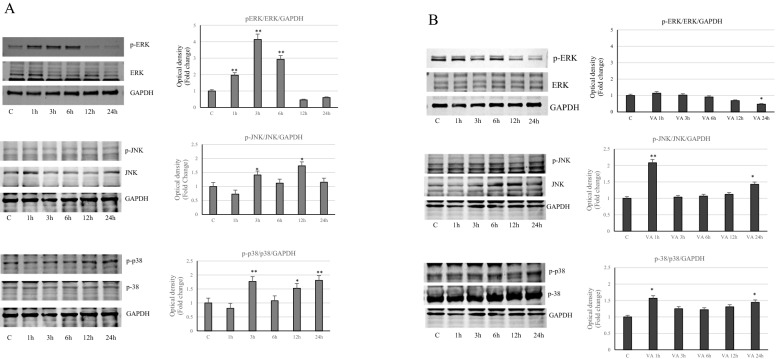
Influence of VA on the MAPK family. Immunoblot of ERK1/2, JNK and p38 protein phosphorylation levels in MCF-7 (**A**) and MDA- MB-231 (**B**) cells at different incubation times (1, 3, 6, 12 and 24 h). GAPDH was used as a protein loading control. Histograms represent the mean ± SD of three experiments in which band intensities were evaluated in terms of arbitrary units of optical density (OD) and expressed as fold change relative to control. *p < 0.05 and **p < 0.005 vs Control (C)

In order to better understand the mechanism of VA action on cell survival, we evaluated the possible involvement of MAP kinases. Since phosphorylation signals are early events, we have assessed them in short times. In this regard, as showed in the Fig. 10A, in MCF-7 cells after a transient up regulation of the phosphoERK1/2, immunoblotting analysis reveals a reduction of phosphorylative events at 12 and 24 h, compared with control cells. Also, in MDA-MB-231 cells, the phospho ERK1/2 decreases with longer treatment times (Fig. 10B). In contrast, we observed increased activation of c-Jun NH2-terminal and p38 (mitogen activated protein) proteins in MCF-7 samples treated with VA, starting from 3 h of treatment. On the other hand, in MDA-MB-231 cells only the phosphorylation of p38 MAPK tends to increase along the treatment (Fig. 10). These results are in accordance with the data obtained from the analysis of proliferative kinetics.

### Role of VA on inflammatory response

**Fig. 11:**
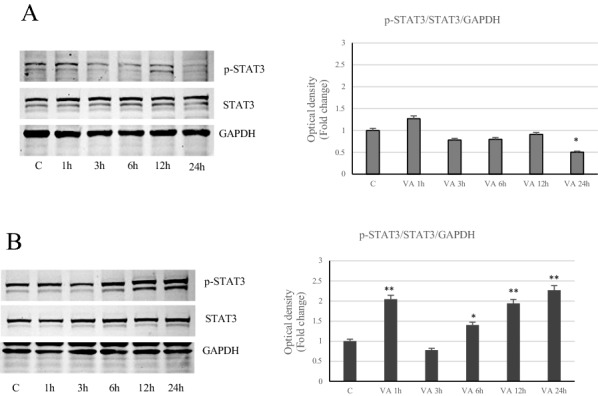
Action of VA on STAT3 in breast cancer cells. Immunoblot of STAT3 protein phosphorylation levels in MCF-7 (**A**) and MDA-MB- 231 (**B**) cells at different incubation times (1, 3, 6, 12 and 24 h). GAPDH was used as a protein loading control. Histograms represent the mean ± SD of three experiments in which band intensities were evaluated in terms of arbitrary units of optical density (OD) and expressed as fold change relative to control. *p < 0.05 and **p < 0.005 vs Control (C)

**Fig. 12:**
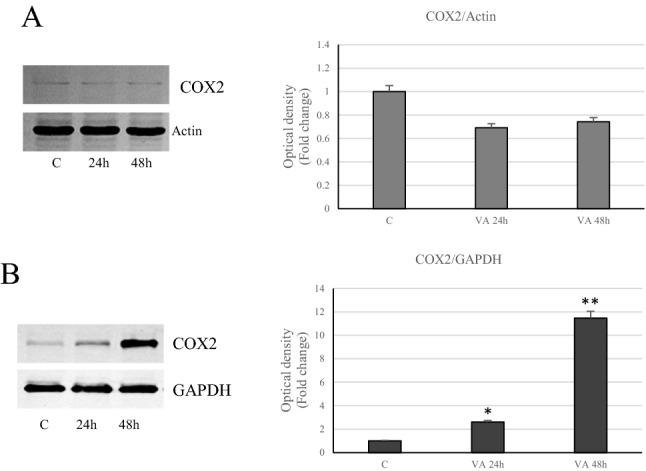
Cox2 levels in VA breast cancer treated-cells. Immunoblots of Cox2 levels in MCF-7 (**A**) and MDA-MB-231 (**B**) cells untreated or treated with valproic acid for 24 and 48 h. GAPDH was used as a protein loading control. Histograms represent the mean ± SD of three separate experiments in which band intensities were evaluated as optical density (OD) and expressed as fold change relative to control *p < 0.05 and **p < 0.005 vs Control (C)

**Fig. 13:**
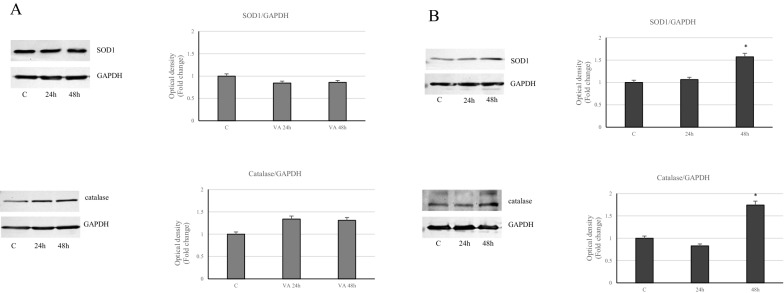
Status of antioxidant enzymes in MCF-7 and MDA-MB-231 cells subjected to VA treatment. Immunoblots of SOD1 and Catalase expressionlevels in MCF-7 (**A**) and MDA-MB-231 (**B**) cells untreated or treated with valproic acid for 24 and 48 h. GAPDH was used as a protein loading control. Histograms represent the mean ± SD of three separate experiments in which band intensities were evaluated as optical density (OD) and expressed as fold change relative to control *p<0.05 vs Control (C).

Different cellular responses induced by VA have been reported in both cell lines, thus we further evaluated the involvement of the inflammatory signals. Our results evidenced that VA was able to significantly activate phospho-STAT3 only in MDA-MB-231 cells (Fig. 11B). In contrast, in MCF-7 cells the same signal tends to decrease under drug treatment, with respect to control, to achieve a more noticeable lowering at the longer time (24 h) (Fig. 11A). Accordingly, Immunoblotting analysis highlighted a down regulation in the expression of the inflammatory enzyme COX2 in VA treated MCF-7 cells, while a clear induction of this enzyme has been observed in MDA-MB-231 cells, particularly at 48 h (Fig. 12). The latter cell type, in which it has been showed a marked increases in ROS, has evidenced a considerable induction of SOD1 and Catalase levels at 48 h (Fig. 13B).Valproate up regulated Catalase expression also in MCF-7 cells (Fig. 13A). Thus in the more aggressive MDA-MB-231 cells, valproate seems to direct the cells towards the inflammatory response.

## Discussion

In breast cancer, epigenetic changes, such as histone acetylation and promoter methylation are considered key factors to address prognosis and therapeutic intervention [33, 34]. Although progresses have been made in the development of cytotoxic regimens, mainly based on DNA-damaging activity, the main obstacles to their successful clinical use are the high toxicity, the side effects and the development of cell resistance to treatment.

Histone deacetylase inhibitors (HDIs), by influencing the expression of genes involved in numerous signaling pathways, including induction of apoptosis, cell cycle arrest and inhibition of angiogenesis, are promising agents as cytostatic element [13]. However, the mechanism of antitumor activity and the specificity of HDIs have not been fully understood. In this study, we aimed to investigate in breast cancer MCF-7 and MDA-MB231 cells the effect of VA on the signaling pathways that regulate cell growth and apoptosis. Our data showed a direct inhibitory action of VA on the growth of breast cancer cells, compared to the normal epithelial phenotype. The growth arrest occurs through cell cycle block in the G0/G1 phase for MCF-7 cells, the reduction of cyclin D1 expression, with the concomitant increase of the cdk inhibitor p21. Differently, valproate in MDA-MB-231 cells induced accumulation of G2/M phase associated with an up-regulation of p21, even in the presence of maintained levels of cyclin B1, important to drives progression into mitosis.

Loss of normal cell cycle control causing aberrant proliferation cells is one of the properties of cancer and novel therapeutic strategies are aimed precisely to target proliferative signals and to address apoptosis [35]. The main regulatory mechanism controlling cell cycle progression is the sequential activation and inactivation of different cyclin-dependent kinases (D, E, B and A) and the activity of cdk inhibitors, which belong to the Cip/Kip families (p21^Cip/WAF1^, p27^Kip1^ and p57^Kip2^) and InK4 (cdk4 inhibitors) families [36]. The cyclin-dependent kinase inhibitor p21 is the principal mediator of cell cycle arrest, as a result of DNA damage, by inactivating G1-phase cyclins/CDKs complexes and maintaining the G2-phase arrest. In this study, we have shown that the VA directs cells towards apoptosis particularly in MCF-7 cells, as documented by using PI and Annexin V double staining.

Indeed, treatment with VA reduces the levels of the anti-apoptotic protein Bcl-2 and increases the expression of pro-apoptotic proteins Bax and Bad, leading to up-regulation of cytochrome C from the outer mitochondrial membrane. Furthermore, in MCF-7 cells, valproate induces the proteolytic form of PARP (poly ADP-ribose polymerase), a crucial target that signals the presence of DNA damage and facilitates its repair. The MDA-MB-231 cells react in a different way to VA treatment, since they show increase of survival protein Bcl2, lowering of Bax levels, together with a sustained activation of phosphorylated Bad, which plays a role in supporting cell survival.

An other important protein involved in inhibition of apoptosis is survivin, which belongs to the IAPs family, able to affect caspase 3 and caspase 7 activities [37]. Survivin is expressed during fetal state but is absent in adult tissue and overexpressed in most tumors, including breast, liver, gastric, colorectal, lung, melanoma and bladder cancers [38–41]. In this study, it has been reported a significat reduction of survivin expression in both cancer-treated cells, confirming a well responsiveness of the cells to the drug, particularly at longer time. The targeted action of VA on this protein is relevant, since its overexpression in tumors causes resistance to cell death. As already reported by other authors, in breast cancer, the aberrant expression of survivin is associated with a worse prognosis and drug resistance [42].

Consistent with these results, there is an increase in ROS production under VA, demonstrating oxidative stress and impaired mitochondrial oxidative metabolism. However, when mitochondrial dysregulation occurs, a reduction of mitochondrial membrane potential and mitochondrial mass, is highlighted, particularly in MCF-7 cells. Furthermore, the same apoptosis induced by VA, as above mentioned, tends to maintain high ROS levels.

Many chemotherapeutical agents for breast cancer promote ROS generation. In fact, Taxanes in breast cancer cells, by leading mitotic block and apoptosis, with Cytochrome C release by mitochondria, support the superoxide formation. Analogously, other drugs such as platinum, anthracyclines, used in therapy for several cancer types, work in the same way [43]. Indeed, VA has been reported to decreases breast cancer cell viability, through inhibition of cell cycle, with the induction of apoptosis [44]. Other authors have reported that VA is able to address apoptosis even in breast cancer stem cells, which are crucial elements for tumor initiation and progression [45].

Suppression of breast cancer cells growth, induced by VA, also involves the phosphorylative pathways of the MAPK family. In fact, we found that valproate, over time, reduces the phosphorylative levels of ERK1/2, kinases primarily involved in the regulation of cell proliferation and differentiation, while it increases the activation of the other two members of the MAPK family, p38 and JNK. Both are traditionally reported as stress responsive MAPKs, since they are activated bystressful stimuli, that induce proliferative arrest, apoptosis and inflammation even though there is a wide variety of overlapping signals to consider.

The increased levels of p38 MAPK have been reported to induces the G1/S checkpoint and to down regulate cyclin D1, important for the S phase transition, as it occurs in MCF7 cells [46, 47]. Similarly, JNK is often considered to be related to its pro-apoptotic activity. Indeed, loss of JNK signaling can enhance tumor progression and induce drug resistance in various types of cancers [48–50].

From the data it emerges that the cytotoxic activity shown in MCF-7 cells treated with VA, is correlated with the triggering of the apoptotic response, with the impairment of mitochondrial function and with the modulation of the MAPK signal. In MDA-MB-231 cells there is no clear overlap of responses with those found in MCF-7 cells. Although there is a considerable ROS production under VA, MDA-MD-231 cells do not show a clear apoptotic response. For this reason, we focused our attention to other signals, such as those related to inflammation [51, 52].

The data revealed the activation of phosphorylated STAT3 and increased levels of the key mediator of inflammation enzyme COX2, only in MDA-MB-231 cells. In support of these observations, there is the up-regulation of Catalase and Sod1, whose levels increased due to the raise of oxidative stress, as occurs in MDA-MB-231 cells.

In this way, the cells attempt to control oxidative stress through increased enzyme expressions and this to minimize the induced injury.

However, it is generally accepted that this occurs in normal cells, where the cellular maintenance of redox homeostasis is controlled by a complex action of antioxidant enzymes. Compared to normal cells, ROS production is elevated in tumors, as there is an increase in metabolic rate, hyperproliferation, angiogenesis condition and frequent genetic instability. The overproduction of ROS in cancer cells is associated with down- regulation of cellular antioxidant enzymes, in order to maintain tumor progression [53].

However, ROS are also able to address apoptosis, to promote cell death and to lead inflammation [54]. Many drugs used in anticancer therapy induce oxidative stress as well apoptosis [55–57] (Additional files 1, 2).

Moreover, our study showed that the proliferative arrest induced by the VA in MDA-MB-231 cells, in which there is a greater production of ROS, compared to MCF-7 cells, involves an inflammatory response supported by the activation of phospho-STAT3 and by the increased levels of COX2. These latest results indicate that in the more aggressive cell phenotype, such as MDA-MB-231 cells, VA has less significant inhibitory effects on tumor progression, than that observed in ER-positive breast cancer cell type.

In fact, other authors have reported that estrogen-sensitive breast cancer cells lines are more responsive to VA than estrogen-insensitive breast cancer cells, suggesting the involvement of different metabolic pathways [58, 59]. Thus, our data showed that VA is able to inhibit cell growth in both MCF-7 and MDA-MB-231 cells, by triggering different responses in a cell type-specific manner.

Overall, the study demonstrates that treatment with VA induces activation of the intrinsic apoptosis pathway, in human breast carcinoma cells ER-positive MCF-7, while in MDA-MB-231 cells other signals linked to inflammation, could be involved.

Our study may contribuite to understand the molecular mechanism through which VA counteractbreast cancer growth and survival. The divergent effects observed in relation to the tumor phenotype suggest the possible implementation of Valproic Acid for the treatment of breast tumor.

## Supplementary Information

**Additional file 1**. Original W.B.

**Additional file 2: Table S1**. Catalog number and diluition of all the antibodies used.

## Data Availability

All data generated or analyzed during this study are included in this published article.

## Abbreviations

ER: Estrogen receptor
PR: Progesteron receptor
HER 2: Human epidermal growth factor receptor 2
SERMS: Selective estrogen receptor modulator
SERDS: Selective estrogen receptor degraders
EGF: Epidermal growth factor
PBS: Phosphate buffered saline
