# Supplementary material for: Valproic acid inhibits cell growth in both MCF-7 and MDA-MB231 cells by triggering different responses in a cell type-specific manner
Source: J Transl Med. 2023 Mar 2;21:165. doi: 10.1186/s12967-023-04015-8 (PMC9983172; doi:10.1186/s12967-023-04015-8)
Supplement: Supplementary file 1 — Additional file 1. Original W.B. [file 12967_2023_4015_MOESM1_ESM.pdf]

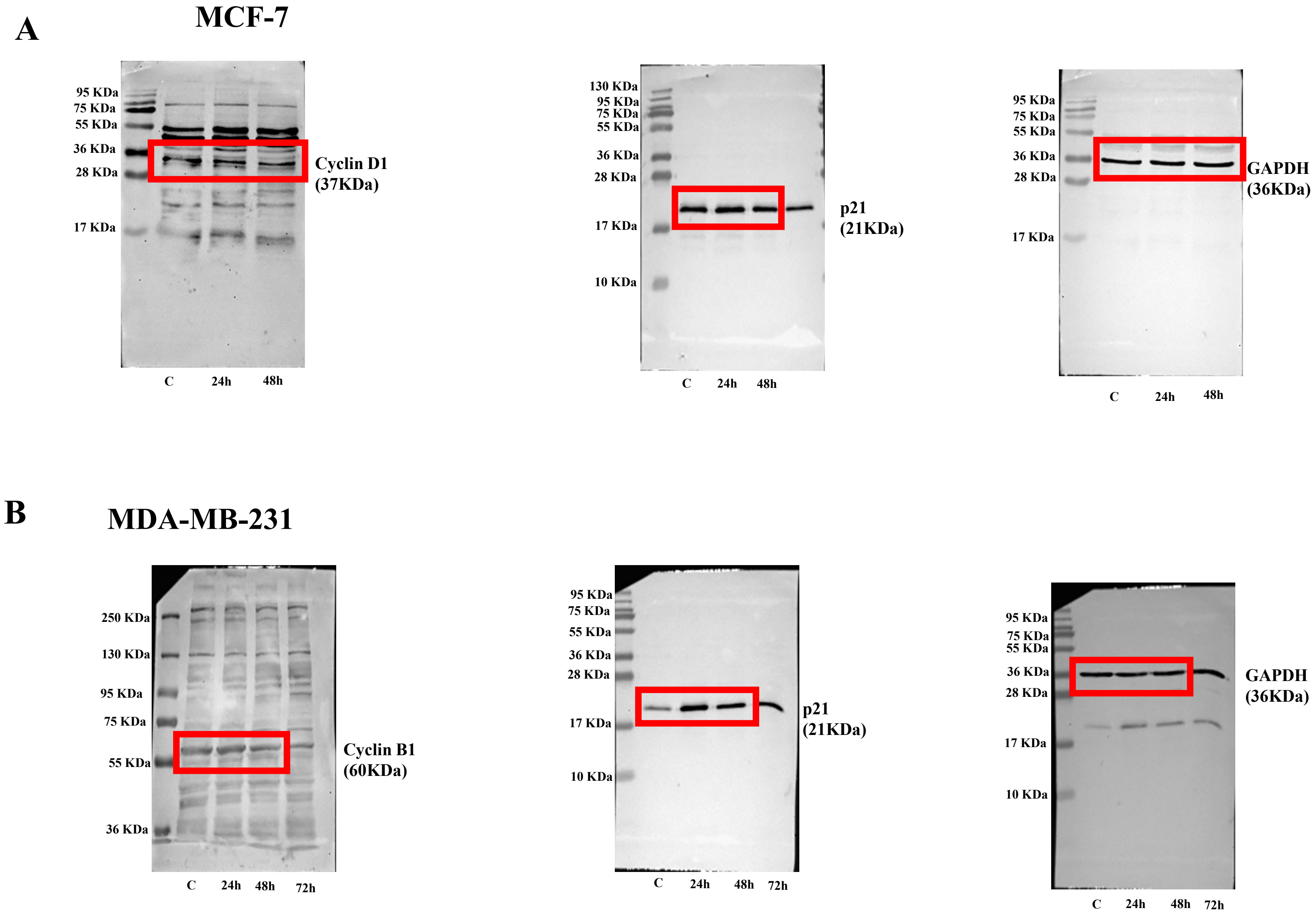
A
MCF-7
95 KDa
75 KDa
55 KDa
36 KDa
28 KDa
17 KDa
Cyclin D1
(37KDa)
C
24h
48h
130 KDa
95 KDa
75 KDa
55 KDa
36 KDa
28 KDa
17 KDa
10 KDa
p21
(21KDa)
C
24h
48h
95 KDa
75 KDa
55 KDa
36 KDa
28 KDa
17 KDa
GAPDH
(36KDa)
C
24h
48h
B
MDA-MB-231
250 KDa
130 KDa
95 KDa
75 KDa
55 KDa
36 KDa
Cyclin B1
(60KDa)
C
24h
48h
72h
95 KDa
75 KDa
55 KDa
36 KDa
28 KDa
17 KDa
10 KDa
p21
(21KDa)
C
24h
48h
72h
95 KDa
75 KDa
55 KDa
36 KDa
28 KDa
17 KDa
10 KDa
GAPDH
(36KDa)
C
24h
48h
72h

Figure 3

MCF-7

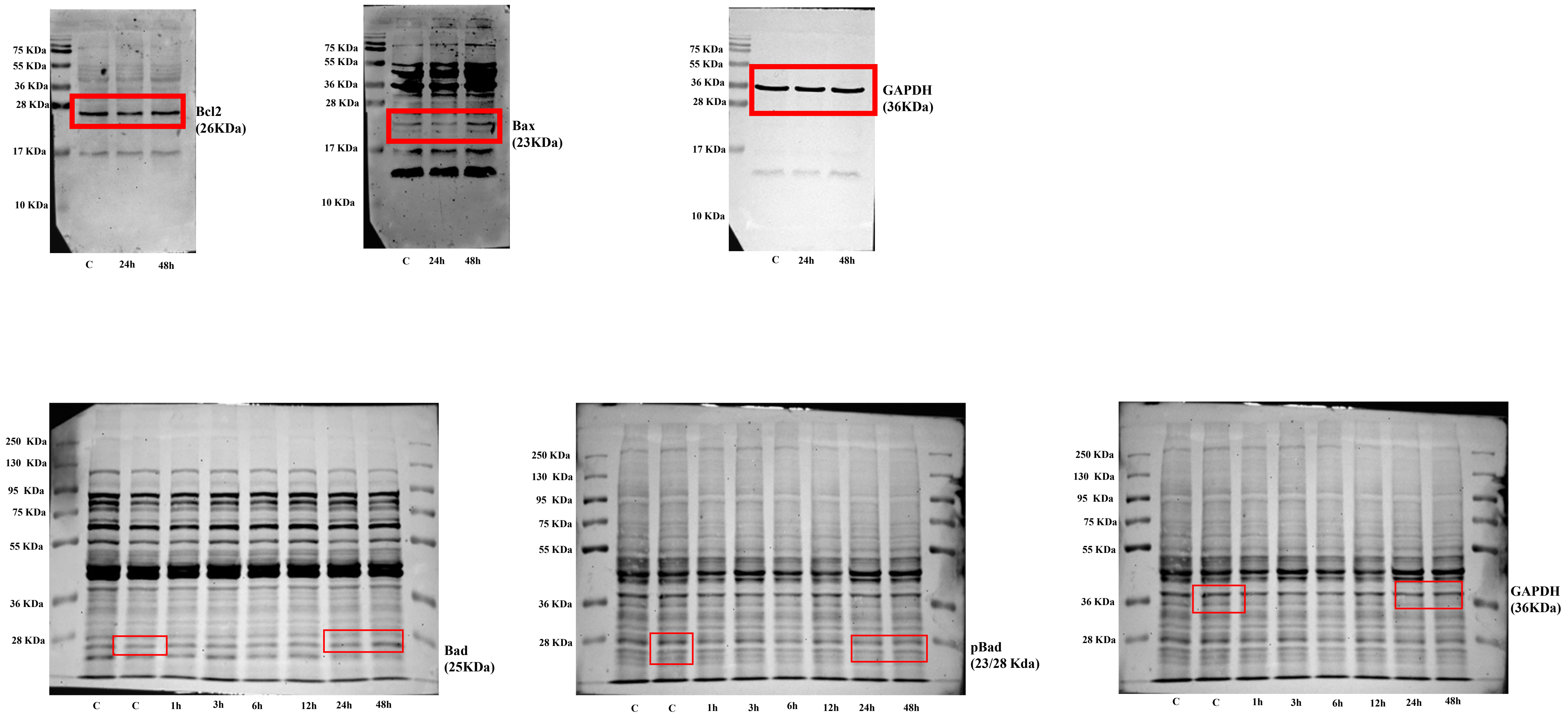

Figure 6

MDA-MB-231

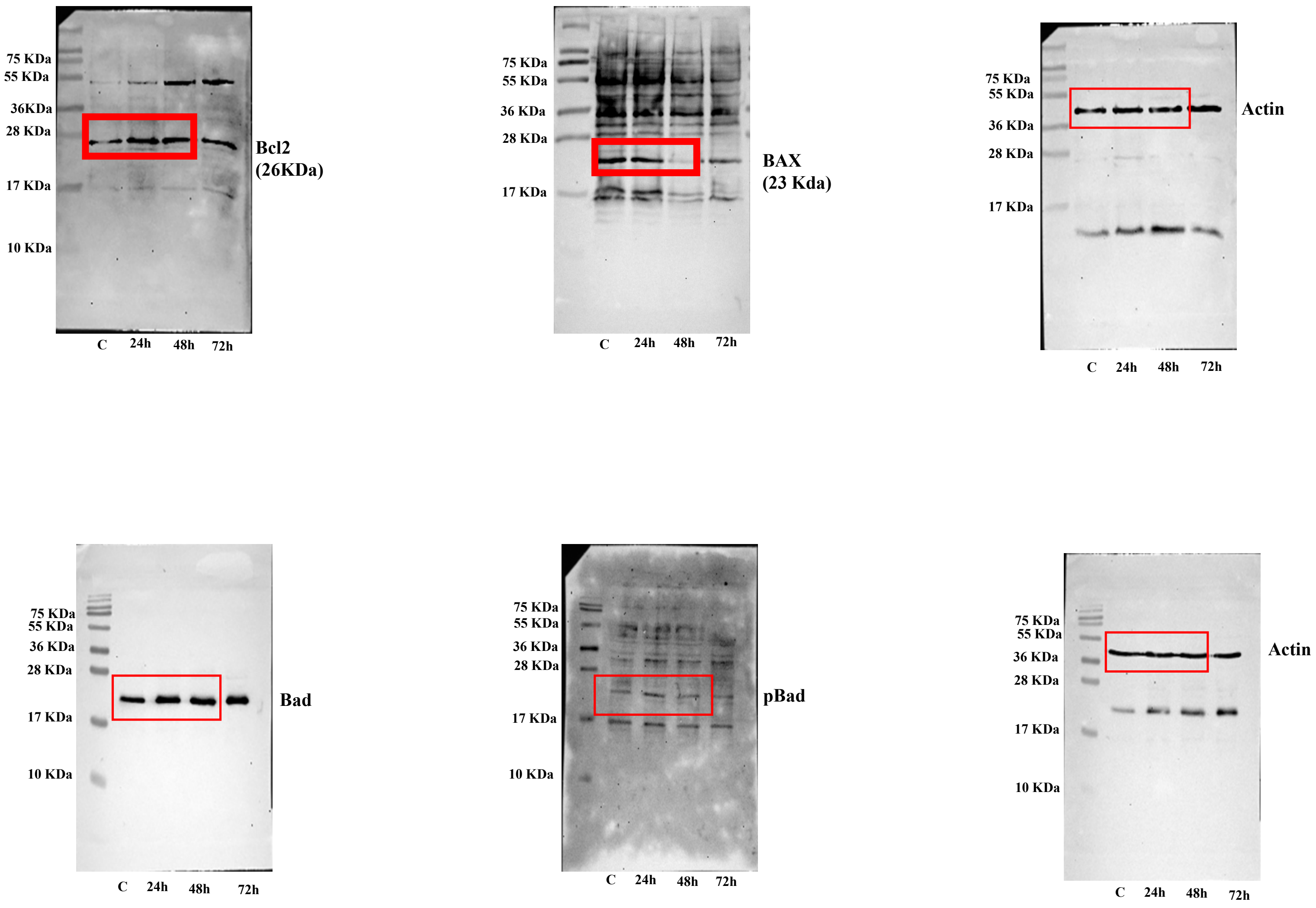

Figure 7

MCF-7

A

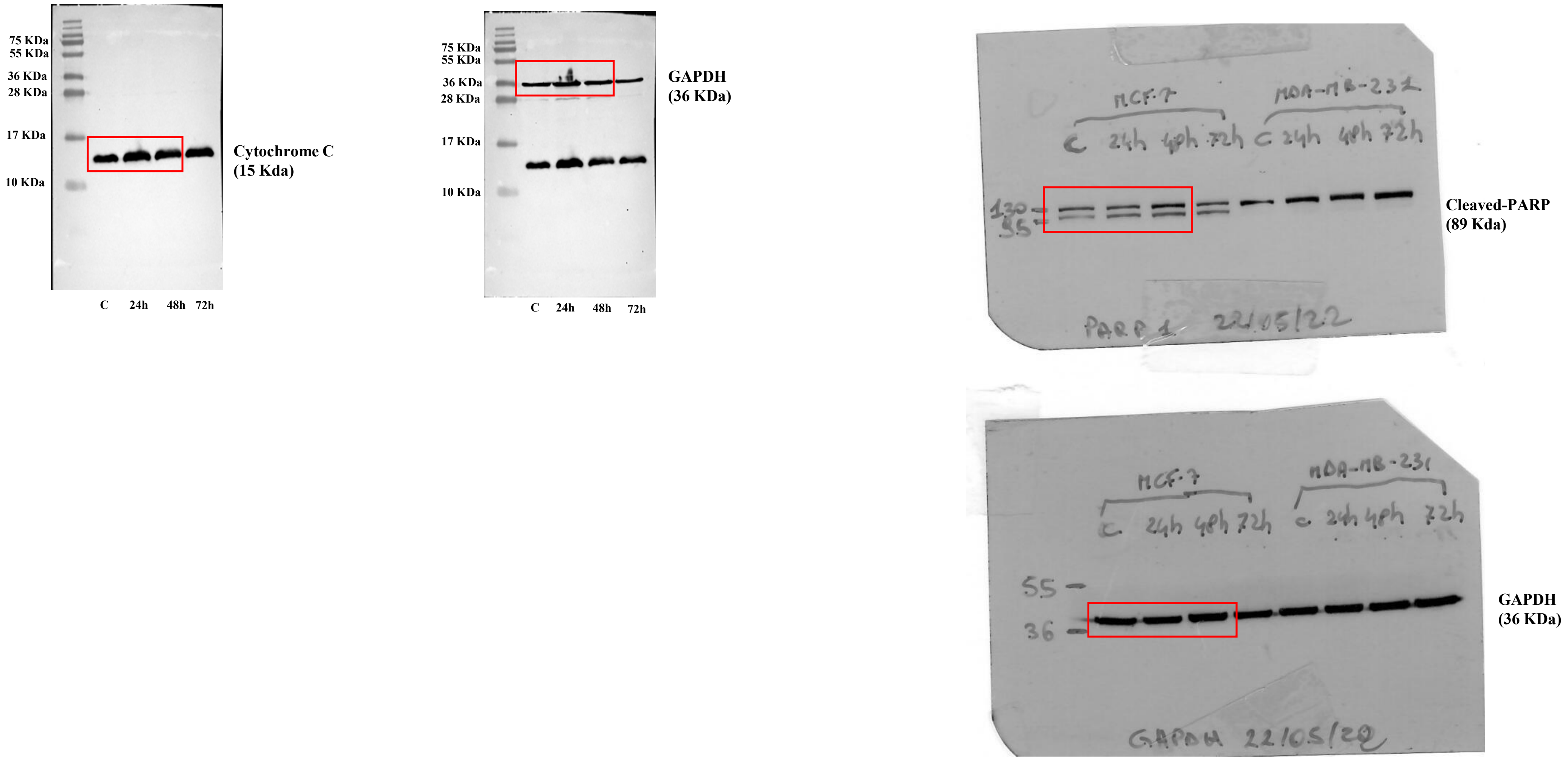

Figure 8 A

MDA-MB-231

B

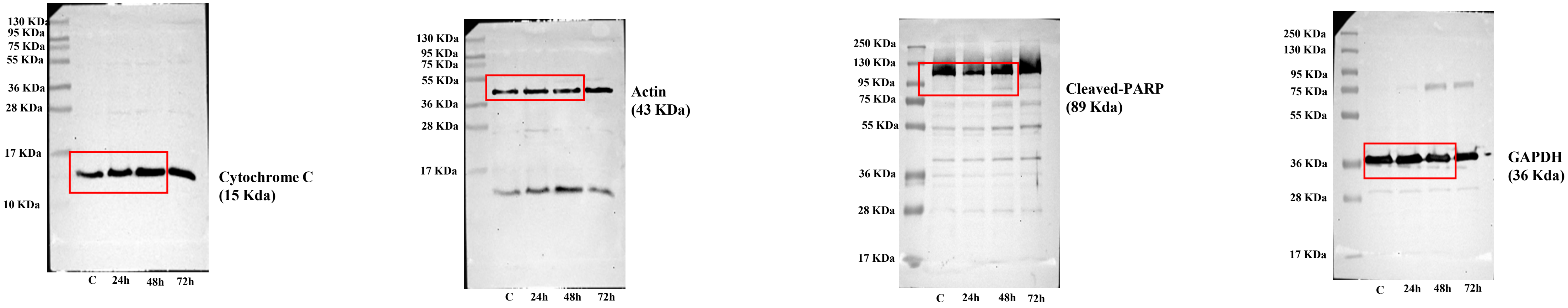

Figure 8 B

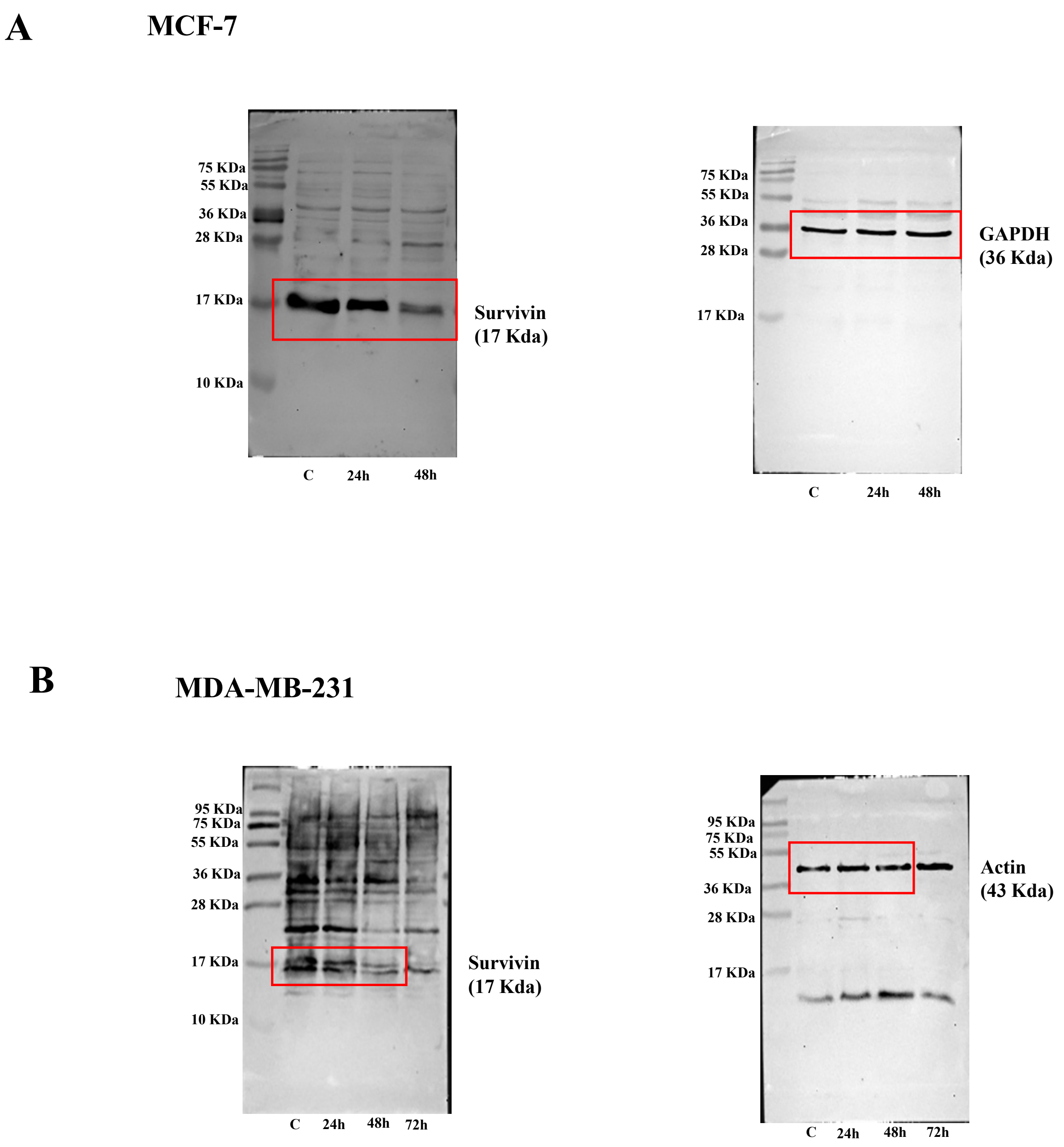
A
MCF-7
75 KDa
55 KDa
36 KDa
28 KDa
17 KDa
10 KDa
Survivin
(17 Kda)
C
24h
48h
75 KDa
55 KDa
36 KDa
28 KDa
17 KDa
GAPDH
(36 Kda)
C
24h
48h
B
MDA-MB-231
95 KDa
75 KDa
55 KDa
36 KDa
28 KDa
17 KDa
10 KDa
Survivin
(17 Kda)
C
24h
48h
72h
95 KDa
75 KDa
55 KDa
36 KDa
28 KDa
17 KDa
Actin
(43 Kda)
C
24h
48h
72h

Figure 9

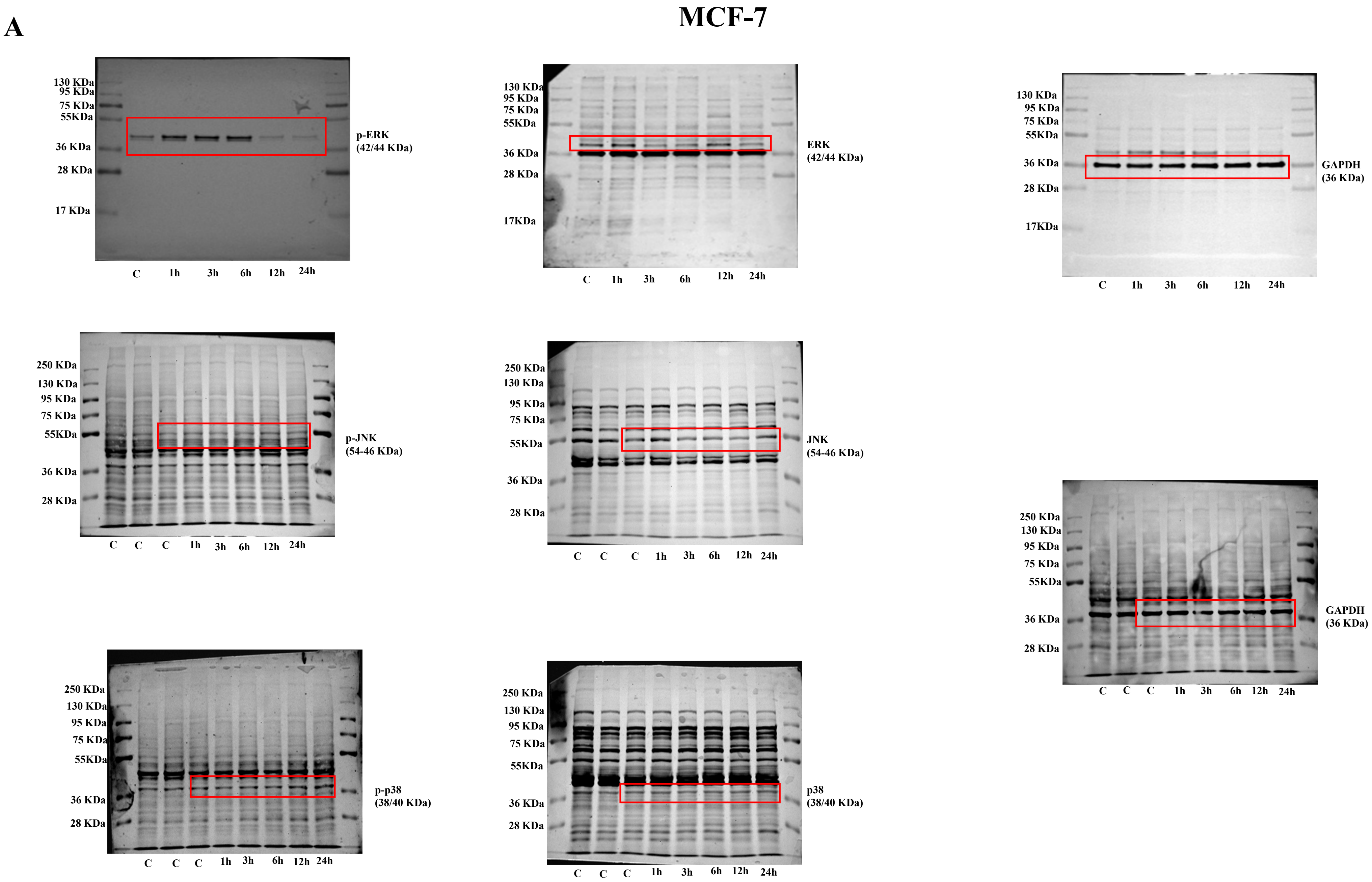
A
MCF-7
130 KDa
95 KDa
75 KDa
55KDa
36 KDa
28 KDa
17 KDa
p-ERK
(42/44 KDa)
C 1h 3h 6h 12h 24h
ERK
(42/44 KDa)
GAPDH
(36 KDa)
250 KDa
p-JNK
(54-46 KDa)
C C C 1h 3h 6h 12h 24h
JNK
(54-46 KDa)
p-p38
(38/40 KDa)
p38
(38/40 KDa)
GAPDH
(36 KDa)

Figure 10 A

B

MDA-MB-231

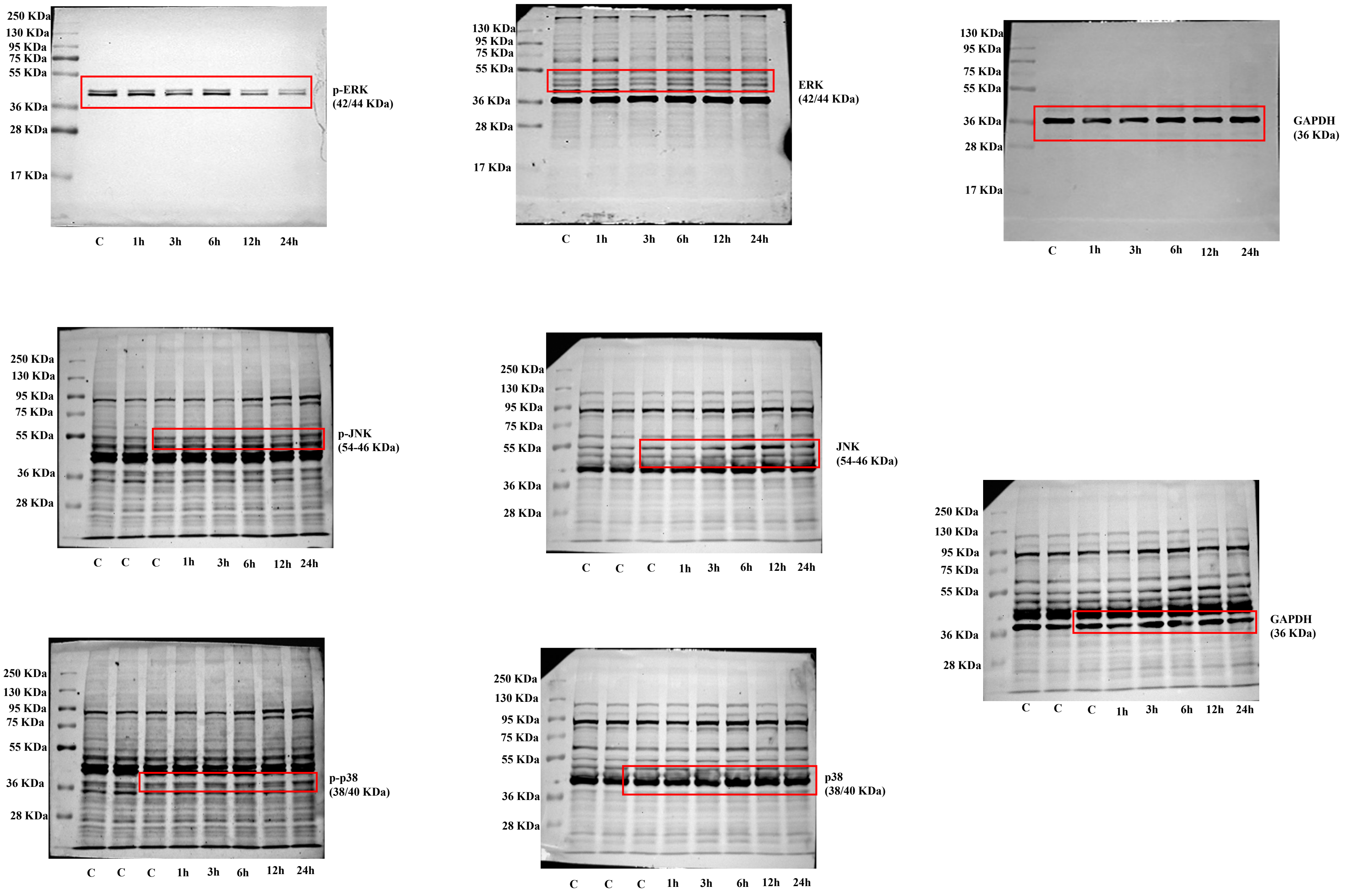

Figure 10 B

A **MCF-7**

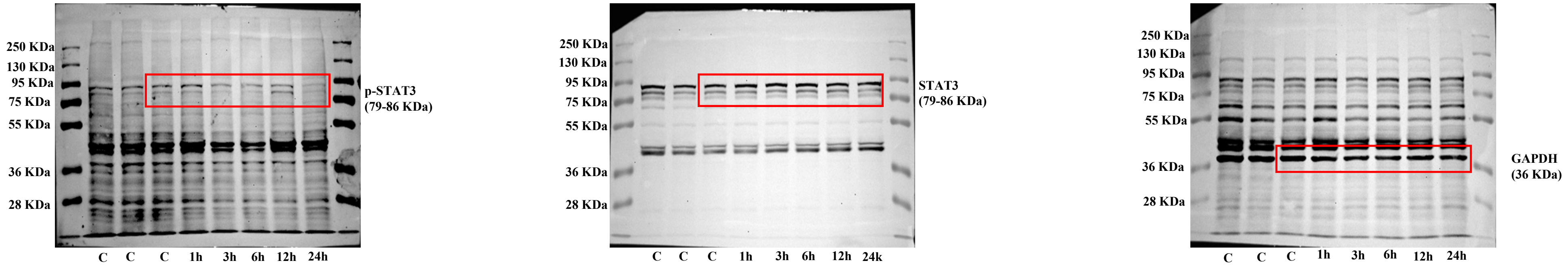

B **MDA-MB-231**

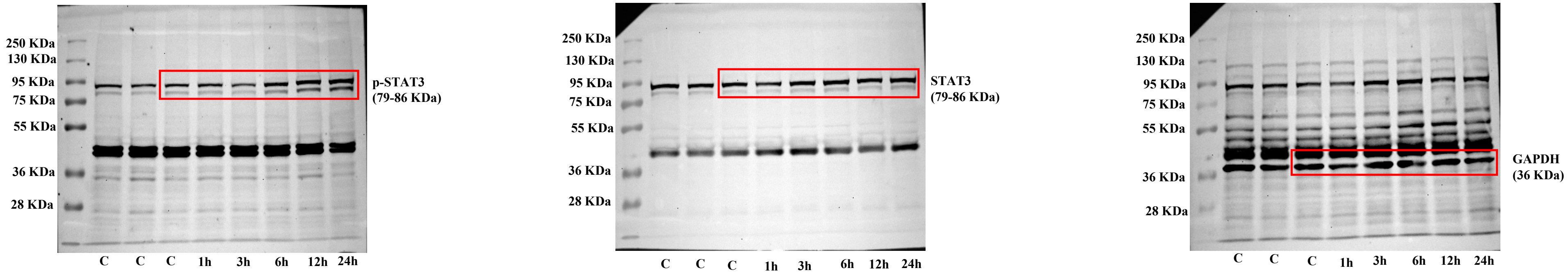

Figure 11

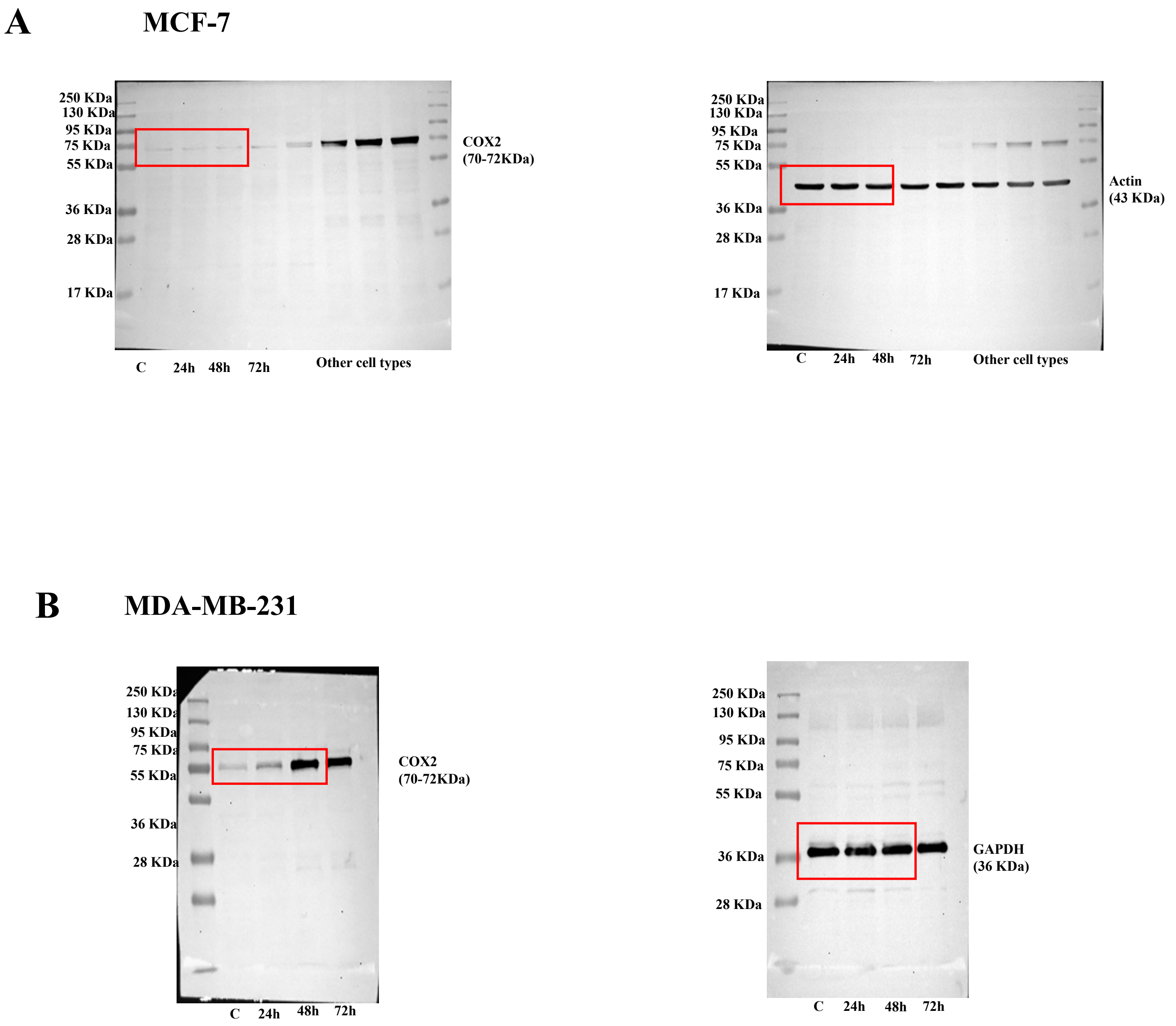
A
MCF-7
250 KDa
130 KDa
95 KDa
75 KDa
55 KDa
36 KDa
28 KDa
17 KDa
COX2
(70-72KDa)
C 24h 48h 72h Other cell types
250 KDa
130 KDa
95 KDa
75 KDa
55 KDa
36 KDa
28 KDa
17 KDa
Actin
(43 KDa)
C 24h 48h 72h Other cell types
B
MDA-MB-231
250 KDa
130 KDa
95 KDa
75 KDa
55 KDa
36 KDa
28 KDa
COX2
(70-72KDa)
C 24h 48h 72h
250 KDa
130 KDa
95 KDa
75 KDa
55 KDa
36 KDa
28 KDa
GAPDH
(36 KDa)
C 24h 48h 72h

Figure 12

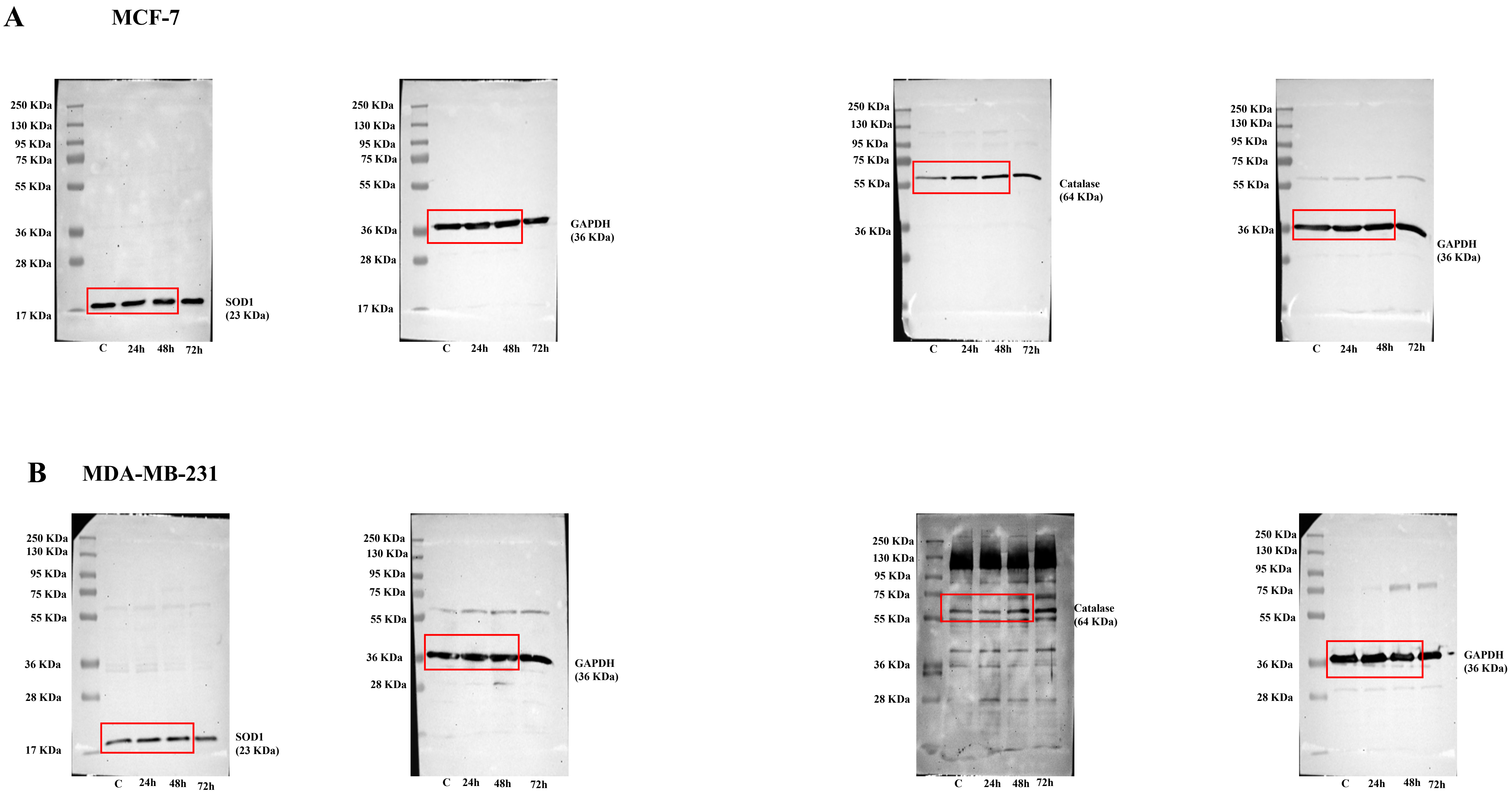
A
MCF-7
250 KDa
130 KDa
95 KDa
75 KDa
55 KDa
36 KDa
28 KDa
17 KDa
SOD1
(23 KDa)
GAPDH
(36 KDa)
Catalase
(64 KDa)
C
24h
48h
72h
B
MDA-MB-231

Figure 13
