# Supplementary material for: Valproic acid inhibits cell growth in both MCF-7 and MDA-MB231 cells by triggering different responses in a cell type-specific manner
Source: J Transl Med. 2023 Mar 2;21:165. doi: 10.1186/s12967-023-04015-8 (PMC9983172; doi:10.1186/s12967-023-04015-8)
Supplement: Supplementary file 2 — Additional file 2: Table S1. Catalog number and diluition of all the antibodies used. [file 12967_2023_4015_MOESM2_ESM.pdf]

Antibody	Catalog Number	Dilution	Manufacturer
Anti-Cyclin D1	MA514512	1:1000	Invitrogen, Thermo Fisher Scientific
Anti-Cyclin B1	PA5120418	1:1000	Invitrogen, Thermo Fisher Scientific
Anti-p21	MA531479	1:1000	Invitrogen, Thermo Fisher Scientific
Anti-p38	AH01202	1:1000	Invitrogen, Thermo Fisher Scientific
Anti-p-ERK	MA515173	1:1000	Invitrogen, Thermo Fisher Scientific
Anti-p-JNK1/2/3	bsm52462R	1:1000	Bios, Massachusetts, USA
Anti-Bax	bsm33279M	1:500	Bios, Massachusetts, USA
Anti-Bcl2	sc-7382	1:500	Santa Cruz Biotechnology, DBA, Milan, Italy
Anti-Bad	sc-8044	1:500	Santa Cruz Biotechnology, DBA, Milan, Italy
Anti-p-Bad	sc-271963	1:500	Santa Cruz Biotechnology, DBA, Milan, Italy
Anti-Cytocrom C	sc-13156	1:500	Santa Cruz Biotechnology, DBA, Milan, Italy
Anti-Survivin	sc-10811	1:500	Santa Cruz Biotechnology, DBA, Milan, Italy
Anti-COX2	sc-19999	1:500	Santa Cruz Biotechnology, DBA, Milan, Italy
Anti-Catalase	sc-271803	1:500	Santa Cruz Biotechnology, DBA, Milan, Italy
Anti-SOD1	sc-8637	1:500	Santa Cruz Biotechnology, DBA, Milan, Italy
Anti-ERK2	sc-154	1:500	Santa Cruz Biotechnology, DBA, Milan, Italy
Anti-Actin	sc-68879	1:500	Santa Cruz Biotechnology, DBA, Milan, Italy
Anti-JNK	9552S	1:1000	Cell Signaling Technology, Euroclone, Milan, Italy
Anti-p-p38	9211S	1:1000	Cell Signaling Technology, Euroclone, Milan, Italy
Anti-pSTAT3	9138S	1:1000	Cell Signaling Technology, Euroclone, Milan, Italy
Anti-STAT3	9139S	1:1000	Cell Signaling Technology, Euroclone, Milan, Italy
Anti-GAPDH	60004-1-Ig	1:1000	ProteinTech

Additional file 2: Table S1. catalog number and diluition of all the antibodies used
